# Molecular detection of *Anaplasma phagocytophilum* in wild carnivores in north-eastern Poland

**DOI:** 10.1186/s13071-019-3734-y

**Published:** 2019-10-07

**Authors:** Tomasz Szewczyk, Joanna Werszko, Anna W. Myczka, Zdzisław Laskowski, Grzegorz Karbowiak

**Affiliations:** Witold Stefański Institute of Parasitology, Polish Academy of Sciences, Twarda 51/55, 00-818 Warsaw, Poland

**Keywords:** *Anaplasma phagocytophilum*, *Vulpes vulpes*, *Martes* sp., *Mustela putorius*, *Nyctereutes procyonoides*, *Meles meles*, Poland

## Abstract

**Background:**

*Anaplasma phagocytophilum* is an obligate parasitic intracellular bacterium. It is the causative agent of granulocytic anaplasmosis, with effects on human and animal health. In Europe, the pathogen is mainly transmitted among a wide range of vertebrate hosts by blood-sucking arthropods. The aim of this study was to determine the presence of *A. phagocytophilum* in wild carnivores, *viz* raccoon dogs (*Nyctereutes procyonoides*), badgers (*Meles meles*), foxes (*Vulpes vulpes*), martens (*Martes* sp.) and European polecats (*Mustela putorius*), using molecular methods.

**Methods:**

In the present study, 174 spleen samples were collected from adult, wild carnivores hunted in the years 2013–2016. A short fragment (383 bp) of the *16S* ribosomal RNA gene partial sequence was used as a marker to identify *A. phagocytophilum* in spleen samples collected from carnivores using nested PCR.

**Results:**

The prevalence of *A. phagocytophilum* in wild carnivores was 31.61% (55/174). Seven sequences of *A*. *phagocytophilum* were generated from two raccoon dogs, two badgers, one marten, one red fox and one European polecat. Six identical nucleotide sequences were obtained from one raccoon dog, two badgers, one marten, one red fox and one European polecat (*A. phagocytophilum* sequences 1: MH328205–MH328209, MH328211), and these were identical to many *A*. *phagocytophilum* sequences in the GenBank database (100% similarity). The second sequence (*A. phagocytophilum* sequence 2: MH328210) obtained from the raccoon dog shared 99.74% identity with *A*. *phagocytophilum* sequence 1.

**Conclusions:**

To our knowledge, this is the first study to use molecular methods to determine the presence of *A*. *phagocytophilum* in wild carnivores, *viz* raccoon dog, badger, marten and European polecat, in Poland. The detected *A*. *phagocytophilum* sequences (1 and 2) were closely related with those of *A*. *phagocytophilum* occurring in a wide range of wild and domestic animals and vectors.

## Background

*Anaplasma phagocytophilum* is a Gram-negative bacterium belonging to the family *Anaplasmataceae* [1]. It is the causative agent of tick-borne fever in ruminants, also known as bovine or ovine granulocytic anaplasmosis, and of human granulocytic anaplasmosis. *Anaplasma phagocytophilum* is typically detected in wild animals (such as rodents, carnivores and ungulates) and domestic animals (such as cattle, goats and sheep) in the natural environment [2–6]. In the northern hemisphere, *A. phagocytophilum* is transmitted mainly by hard ticks of the genus *Ixodes* [7, 8]. Species of other tick genera, such as *Dermacentor* spp. and *Rhipicephalus* spp., can also serve as biological vectors; however, their significance currently remains unknown. In addition, while *A. phagocytophilum* is known to demonstrate transstadial transmission in tick vectors, transovarial transmission has not been observed [7].

*Anaplasma phagocytophilum* has also been detected in other arthropods such as blood-sucking flies, specifically deer flies (*Lipoptena cervi*) and horse flies (Diptera: Tabanidae) [9]. In addition, wild carnivores can also play a key role in pathogen transmission between wildlife, domestic animals and, possibly, humans. The growing degree of contact between free-living animals, domestic animals and human populations due to climate change and population growth has resulted in an elevated risk of human and bovine anaplasmosis outbreaks [10].

*Anaplasma phagocytophilum* has been detected by molecular methods in many carnivore species. In red foxes, it has been detected in Italy [11, 12], Germany [13], the Netherlands [14], Romania [15], Hungary [16], Switzerland [17], the Czech Republic [18, 19] and Austria [20]. In raccoon dogs, *A*. *phagocytophilum* has been detected in Germany [13] and in South Korea [21].

Knowledge about *A. phagocytophilum* infection in badgers, polecats and martens is still limited. It has been detected in European polecats (*Mustela putorius*) in Germany [22] and in the Netherlands [23], as well as in badgers (*Meles meles*) in the Netherlands and Spain [23, 24]. It has also been identified among pine martens (*Martes martes*) in Belgium. [23].

With the exception of foxes, data regarding the levels of *A. phagocytophilum* infection in many wild carnivores, such as raccoon dogs, European polecats, martens and badgers, is scarce in Poland [25]. While the majority of these carnivores are native to central Europe, the raccoon dog (*Nyctereutes procyonoides*) is an introduced species from Asia. In Poland, they were first identified in the Białowieża Primeval Forest in 1955 [26]; they are currently widespread throughout central Europe, and their range is extending into western Europe [27].

These wild carnivores could potentially act as vectors of *A*. *phagocytophilum* in the natural environment [8]. The aim of the present study was therefore to determine the prevalence of *A*. *phagocytophilum* in five species of predatory wild animals in Poland.

## Methods

Spleen samples were collected from 174 wild carnivores [68 raccoon dogs: 28 females (F)/40 males (M); 49 badgers: 21F/28M; 29 foxes: 13F/16M; 24 martens: 7F/17M; 4 polecats: 2F/2M] hunted in Poland around the Głęboki Bród Forest District (53°98′N, 23°29′E) during the years 2013–2016. These were stored at −20 °C until further processing. DNA was isolated from the middle part of the spleen using a Genomic Mini AX Tissue kit (A&A Biotechnology, Gdynia, Poland) and according to the manufacturer’s instructions.

Molecular detection of *A*. *phagocytophilum* was based on semi-nested PCR amplification of the partial *16S* rRNA gene according to Werszko et al. [9]. Three primers were used to molecular detection of *A*. *phagocytophilum*: A480F, A520F and A900R. The first PCR reaction was performed with primers A480F and A900R. For the second reaction (semi nested PCR), 1 μl of the first reaction product and primers A520F and A900R were used. PCR reactions were conducted in 50 µl of reaction mixture containing the following: 40 μl of deionized water in first reaction (41 μl in second), 1 μl of a 25 mM solution of MgCl_2_, 0.5 μl of Allegro *Taq* DNA Polymerase (5 U/μl; Novazym, Poznań, Poland), 0.5 μl of dNTPmix (10 mM), 5 μl of 10× *Taq* DNA Polymerase Buffer (with 25 mM MgCl_2_), 0.5 μl of each primer (20 pmol/μl), 2 μl of template DNA in the first reaction and 1 μl of first reaction PCR product in the second reaction. DNA isolated from red deer (*Cervus elaphus*) infected with *A. phagocytophilum* (GenBank: GQ450278) was used as the positive control. Nuclease-free water was added to the PCR mix as a negative control. The PCR reaction was performed in an automated DNA Engine PTC-200 Peltier Thermal Cycler (BioRad, Hercules, CA, USA). The thermocycling profile was as follows: denaturation at 92 °C for 2 min, followed by 35 cycles with 30 s denaturation at 94 °C, 10 s primer annealing at 60 °C, and 1 min at 72 °C for primer extension, with a final extension step of 5 min at 72 °C.

The PCR products were subjected to electrophoresis (Bio-Rad Power Pac Basic 85 V, 45 min) on a 1.0% agarose gel stained with ethidium bromide and visualized using ChemiDoc, MP Lab software (Imagine, BioRad); a Nova 100 bp DNA Ladder (Novazym, Poznań, Poland) was used for comparison. The PCR amplicons were purified using a QIAEX II Gel Extraction Kit (Qiagen, Hilden, Germany), sequenced by Genomed (Warszawa, Poland) and assembled into contigs using ContigExpress, Vector NTI Advance v.11.0 (Invitrogen Life Technologies, New York, NY, USA). The obtained sequences were compared with sequences available on GenBank using the Basic Local Alignment Search Tool (BLAST). Statistical analyses were performed using the Chi-square test in GraphPad online (https://www.graphpad.com).

## Results

The overall prevalence of *A. phagocytophilum* infection was 31.60% (55/174); however, among all tested groups, the prevalence of infection varied according to the host species. The highest prevalence was observed in martens (41.70%; 10/24), followed by raccoon dogs (35.30%; 24/68) and foxes (34.48%; 10/29), with the lowest prevalence observed in badgers (18.70%; 9/49). *Anaplasma phagocytophilum* was detected in two of four investigated European polecats. The prevalence of *A*. *phagocytophilum* was significantly higher in martens than in badgers (*χ*^2^ = 4.5422, *df* = 1, *P* = 0.033; 95% CI: 0.16–0.36). The prevalence in raccoon dogs was significantly higher than in badgers (*χ*^2^ = 4.0295, *df* = 1, *P* = 0.045; 95% CI: 0.20–0.36). During the tests, a large difference was observed between the first and the second reaction in nested PCR. After the first reaction only 4 samples were positive in all tested animals (2.30%). After the second amplification step, 55 samples out of 174 were positive (31.60%).

Seven sequences were obtained from *A*. *phagocytophilum* PCR-positive samples and submitted to the GenBank database (from two raccoon dogs, two badgers, one marten, one red fox and one European polecat). Six sequences (*Anaplasma phagocytophilum* sequence 1: MH328205-MH328209, MH328211) were found to be identical (100% similarity) with many *A*. *phagocytophilum* sequences obtained from Europe, Asia, Africa and North America. *Anaplasma phagocytophilum* sequences were obtained from a wide range of hosts: human, dog, raccoon dog, fox, sheep, black-striped field mouse, northern red-backed vole, tick, cow, goat and horse. More detailed data are shown in Table 1. *Anaplasma phagocytophilum* sequence 2 was highly similar to all examples of *A*. *phagocytophilum* sequence 1 obtained in this study (99.74% identity) and differed only by one nucleotide from sequence 1.

**Table 1:** Examples of *A. phagocytophilum* sequences with 100% similarity from the GenBank database found using the Basic Local Alignment Search Tool (BLAST)

Host	Country of isolation	GenBank ID
Human (*Homo sapiens*)	South Korea	MH482862
	South Korea	KP306518-KP306520
	Austria	KT454992
Dog (*Canis lupus familiaris*)	Japan	LC334014
	Croatia	KY114936
	Austria	JX173652
	Germany	JX173651
	South Africa	MK814402-MK814406
Shelter cat (*Felis catus*)	South Korea	KR021165
Raccoon dog (*Nyctereutes procyonoides*)	South Korea	KY458570, KY458571
Fox (*Vulpes vulpes*)	Switzerland	KX180948
Horse (*Equus caballus*)	Sweden	AY527213
Cow (*Bos taurus taurus*)	Turkey	KP745629
Sheep (*Ovis aries*)	Norway	CP015376
Black-striped field mouse (*Apodemus agrarius*)	South Korea	KR611718, KR611719
	China	GQ412337
	China	DQ342324
Bank vole (*Clethrionomys glareolus*)	Russia	AY094352, AY094353
	UK	AY082656
Northern red-backed vole (*Myodes rutilus*)	Russia	HQ630622
Kachin red-backer vole (*Eothenomys cachinus*)	China	FJ968660-FJ968662
Tick (*Ixodes ricinus*)	Estonia	HQ629922
	Russia	HQ629911, HQ629912
	Belarus	HQ629914, HQ629915
Tick (*Ixodes pacificus*)	USA	KP276588
Tick (*Ixodes persulcatus*)	Russia	HM366579-HM366584
	China	AF486636
Tick (*Ixodes ovatus*)	Japan	AY969012
Tick (*Haemaphysalis longicornis*)	China	KF569908, KF569909
	South Korea	GU064898, GU064899

## Discussion

Studies performed in Europe confirm that a wide range of mammals can serve as competent animal reservoirs for *A. phagocytophilum*, and that the composition of this reservoir varies according to geographical regions [8, 28]. The high prevalence of *A*. *phagocytophilum* infection in the tested animals provides compelling evidence for the involvement of wild carnivores in the enzootic cycle. So far in Europe, *A*. *phagocytophilum* has been detected in red foxes in Germany (8.20%), Italy (16.60%) and Hungary (12.50%) [11, 13, 16]. It has also been detected in Poland, but only with an infection rate of 2.70% [25]. The higher prevalence identified in the present study (34.48%) may be associated with infection rate in the vectors [29], or the geographical distribution: the previous study was performed in central Poland, whereas the present study was performed in the north-east. Additionally, the prevalence was found to be 2.7% in the earlier study in Poland [25] which is close to prevalence levels obtained after the first PCR reaction in presented study. *Anaplasma phagocytophilum* can be detected in many various organs such as spleen, lung and liver tissue [4, 12, 13]. Our present findings confirm that semi-nested PCR offers a high level of accuracy in detecting *Anaplasma* in spleen samples, and that it is more sensitive than standard PCR: while the detected prevalence was only 4/174 (2.30%) in all tested animals after the first reaction, the detection level was increased to 55/174 (31.60%) after the second stage.

Foxes are well adapted to the urban environment and are accustomed to the presence of humans. In addition, their frequent exposure to tick bites and their potential to act as reservoirs or maintenance hosts for pathogens infecting humans and domestic animals highlights their importance in public health [15]. The prevalence of *A*. *phagocytophilum* observed in raccoon dogs in Poland (35.30%) is higher than in Germany (23.00%) [13]. Interestingly, while Han et al. [21] and Kang et al. [30] reported the prevalence of *A. phagocytophilum* infection in this host to be 1.00% in Korea, Hildebrandt et al. [31] reported no infection in raccoon dogs in western Poland.

According to the present findings, 18.70% of the tested badgers in north-eastern Poland displayed *A*. *phagocytophilum* infection; however, Garcia-Pérez et al. [24] reported a prevalence of 1.50% among badgers in Spain, and Hofmeester et al. [23] found it to be 1.80% in the Netherlands. While *Anaplasma* infection was found in two of the four tested European polecats in the present study, previous studies have found the prevalence to be 4.30% in Germany and 4.90% in the Netherlands [22, 23]. Similarly, *A. phagocytophilum* was found to be more common in pine martens (*Martes martes*) in the present study (41.70%) than in Belgium (22.00%) [23].

## Conclusions

All five tested species of carnivore may serve as suitable reservoir hosts in the ecology of *Anaplasma phagocytophilum* in the natural environment. Most isolates obtained in this study were identical to *A*. *phagocytophilum* sequences deposited in GenBank. Our findings represent the first detection of *A. phagocytophilum* in badgers, raccoon dogs, martens and polecats from Poland. The nested PCR used in the present study was more sensitive to detection of *A. phagocytophilum* than standard PCR for spleen tissue samples.

## Data Availability

The data supporting the conclusions of this article are included within the article. The sequences were submitted to the GenBank database under the accession numbers MH328205-MH328211 (*Anaplasma phagocytophilum*).

## Abbreviations

rDNA: ribosomal DNA
PCR: polymerase chain reaction
BLAST: basic local alignment search tool
